# Pomegranate Peel Extract Decreases Plaque Necrosis and Advanced Atherosclerosis Progression in *Apoe* ^*-/-*^ Mice

**DOI:** 10.3389/fphar.2022.888300

**Published:** 2022-06-01

**Authors:** Vijayprakash Manickam, Umesh Kumar Dhawan, Damanpreet Singh, Mahesh Gupta, Manikandan Subramanian

**Affiliations:** ^1^ CSIR—Institute of Genomics and Integrative Biology, New Delhi, India; ^2^ Academy of Scientific and Innovative Research (AcSIR), Ghaziabad, India; ^3^ Pharmacology and Toxicology Laboratory, Dietetics and Nutrition Technology Division, CSIR-Institute of Himalayan Bioresource Technology, Palampur, India; ^4^ Food and Nutraceutical Laboratory, Dietetics and Nutrition Technology Division, CSIR-Institute of Himalayan Bioresource Technology, Palampur, India; ^5^ Barts and The London School of Medicine and Dentistry, Queen Mary University of London, London, United Kingdom

**Keywords:** atherosclerosis, pomegranate (*Punica granatum* L.), efferocytosis, vulnerable plaque, MERTK

## Abstract

Atherosclerosis is a chronic lipid-driven inflammatory condition of the arteries and is a leading cause of stroke, myocardial infarction, and other peripheral arterial diseases. Plant products rich in polyphenols such as pomegranate juice and peel extract are known to have beneficial effects in suppressing atherogenesis. However, the mechanism of action and its effect on advanced atherosclerosis progression which results in adverse clinical outcomes are not well understood. Herein, we use a standardized hydroethanolic extract of *Punica granatum* (pomegranate) peel in the *Apoe* ^*-/-*^ a murine model of advanced atherosclerosis. It was observed that the pomegranate peel extract fed mice have decreased plaque necrosis and elevated lesional collagen content which was associated with a favorable metabolic profile including lowering of blood glucose, cholesterol, and triglyceride. The decrease in plaque necrosis was linked with increased lesional macrophage efferocytosis efficiency which was associated with enhanced expression of the efferocytosis receptor Mertk. Using *in vitro* studies, we show that pomegranate peel extract blocks the shedding of Mertk and preserves macrophage efferocytosis efficiency. These data identify a novel mechanism by which pomegranate peel extract promotes the resolution of inflammation in atherosclerosis.

## Introduction

Atherosclerosis is the foremost cause of cardiovascular morbidity and mortality resulting in a stroke, myocardial infarction, and other peripheral arterial diseases (Virani et al., 2021). Cholesterol lowering therapies, particularly statins, have been moderately successful in lowering mortality from atherothrombotic complications (Rodriguez et al., 2017). Nevertheless, atherosclerosis presents a significant medical and economic burden. It is now well understood that atherosclerosis progression is mediated by a combination of dyslipidemia, oxidative stress, and impairments in inflammation resolution responses (Kasikara et al., 2018). Thus, there is a need for novel treatment strategies that promote inflammation resolution and suppress oxidative stress that can be used along with cholesterol lowering drugs like statins and PCSK9 inhibitors.

Plant products that are rich in polyphenols have been shown potent anti-inflammatory and antioxidant activities. The consumption of polyphenols-rich plant products has been associated with beneficial cardioprotective and vasculoprotective effects in preclinical and clinical studies (Kirichenko et al., 2020). In this context, the consumption of pomegranate juice or extract from pomegranate peel, flower, or seeds has been demonstrated to promote a favorable metabolic profile and lowering of cardiovascular risk factors (Aviram et al., 2004; Wang et al., 2018). Similarly, administration of pomegranate juice to atherosclerosis-prone animals has been shown to decrease lesion size in the aortic root and other major vascular beds (Aviram et al., 2000). However, the changes to the atherosclerotic lesional cellular composition, inflammation, plaque necrosis, and collagen deposition are not known. This is critical since plaques that rupture and lead to atherothrombotic complications are often characterized by heightened inflammation, plaque necrosis, thinning of the fibrous cap, and reduced collagen (Finn et al., 2010). The expansion of the necrotic core in the atherosclerotic plaque is driven by a defect in the clearance of apoptotic cells (efferocytosis) by lesional phagocytes (Yurdagul et al., 2018). Interestingly, therapeutic strategies that increase lesional macrophage efferocytosis efficiency have been found to stimulate the resolution of inflammation and stabilization of atherosclerotic plaque in several pre-clinical animal models of atherosclerosis (Dhawan et al., 2021). Recently, the administration of the anti-CD47 antibody, which increases lesional efferocytosis in murine atherosclerosis models (Kojima et al., 2016), was demonstrated to decrease lesional inflammation in patients with coronary artery disease (Jarr et al., 2021). In this context, the effect of pomegranate extracts on key cellular and molecular mechanisms that drive advanced atherosclerotic plaque progression is currently unknown.

While all components of pomegranate seem to provide vasculoprotective effects, pomegranate peel has shown the highest antioxidant capacity and polyphenol content (Li et al., 2006). Since pomegranate peel is considered an agricultural waste product, the development of nutraceuticals utilizing pomegranate peel is an attractive strategy to provide value for money and promote waste reduction and sustainable production.

In this context, herein we report that a standardized hydroethanolic extract of pomegranate peel that is enriched in polyphenols prevented advanced atherosclerosis progression in western type diet fed *Apoe* ^*-/-*^ mice. Specifically, we demonstrate that pomegranate peel extract fed mice have significant favorable cellular remodeling of plaque and are associated with decreased plaque necrosis and enhanced collagen deposition. We also show that the decrease in plaque necrosis in PPE-fed mice is associated with improved lesional macrophage-mediated clearance of dead cells (efferocytosis). Mechanistically, we demonstrate that PPE prevents the cleavage of the efferocytosis receptor Mertk on the macrophages possibly by lowering cellular oxidative stress.

## Materials and Methods

### Material

The pomegranate variety Ganesh was procured in Palampur (H.P.), India (32.1109° N, 76.5363° E). All reference standards used were obtained from Sigma-Aldrich. The total dietary fiber kit (K-TDFR, 200A) was procured from Megazyme (Wicklow, Ireland).

### Sample Preparation

The peels were separated from fresh pomegranate and were dried for 48 h using a hot air drier at 60°C (Macro Scientific Work, Pvt, Ltd. India) followed by grinding to a fine powder which was stored in a cool and dry place. The processed powder was used for hydroethanolic extraction, characterization, and analysis.

### Extraction and Polyphenol Quantification

The bioactive from pomegranate peel were extracted using different hydroethanolic solvent concentrations from 50 to 100%. Powdered peel was extracted using hydroethanolic solvent at a 1:10 w/v ratio for 1 h at 80°C in a hot water bath using different ratios of ethanol. Furthermore, the mass was filtered using Whatman filter paper and concentrated on a rota evaporator (BUCHI F-305). Finally, the concentrated extract was lyophilized (Labconco Free Zone 6 Plus, United States) to obtain the dry extract.

Total phenolic content was analyzed using the Folin-Ciocateu reagent (Bhatt et al., 2021). In brief, 1 mg/ml of extract was mixed with 0.5 ml of Folin-Ciocateu reagent (1 N) followed by the addition of 7% w/v saturated sodium bicarbonate. The mixture was incubated at room temperature (25°C) for 30 min. The absorbance at 730 nm was recorded and the data were expressed as mg of gallic acid equivalents (GAE) per gram of dry sample.

Total flavonoid content was determined as described earlier (Bhatt et al., 2020). Briefly, the extracts were mixed with sodium nitrite (5%) and aluminum chloride and reacted with sodium hydroxide (1 N). The absorbance at 730 nm was measured and the results were expressed as mg of rutin equivalents per gram of dry sample.

### Proximate Analysis

Proximate analysis of peel sample with maximum phenolic content *i.e.* 60% hydroethanolic extraction was conducted as described earlier (Bhatt et al., 2021). Total dietary fiber content was estimated using a Megazyme kit.

### UPLC Analysis of Phenolics

The quantification of phenolic compounds was carried out on a Shimadzu LCMS-2020 system equipped with a PDM30A detector. Separation was achieved on BEH-C18 (1.7 μm) column, fitted with a suitable guard column. The mobile phase consisted of 0.1% formic acid in water (A) and methanol (B). The column temperature was set at 30°C. Standards and samples were eluted at a flow rate of 0.24 ml/min using a gradient system. Compounds were identified based on retention time, co-injections, and spectral matching with standards that were procured from Sigma Aldrich. The following standards were used: gallic acid (Cat# 147915), chlorogenic acid (Cat# C3878), p-coumaric acid (Cat# C9008), caffeic acid (C0625), and punicalagin A and B (Cat# P0023).

### Animal Maintenance and Experimental Design

*Apoe* ^*-/-*^ mice (Stock # 002052) were bred and maintained as homozygotes in a temperature- and humidity-controlled facility with a 12 h light/dark cycle. C57BL6 mice were bred in-house. Commercially available pelleted standard chow diet and autoclaved water was supplied *ad libitum*. All animal experiments were approved by the Institutional Animal Ethics Committee of CSIR-Institute of Genomics and Integrative Biology, New Delhi, India.

For atherosclerosis studies, eight-week-old male and female *Apoe* ^*-/-*^ mice were randomized into two groups (5 mice/group per sex). The animals were fed ad libitum with a high fat high cholesterol western type diet (Research Diets, New Brunswick, NJ, United States, Research Diets no. D12079B) and were simultaneously administered 200 mg/kg PPE or vehicle (water) *via* daily oral gavage for 12 weeks. Daily food consumption was monitored by weighing the residual food in the cage hopper. After euthanasia, blood was collected by intracardiac puncture, and the isolated plasma was used for measuring levels of AST, ALT, and creatinine using commercially available calorimetry-based kits (Cat # MAK055, MAK052, and MAK080 respectively; Sigma-Aldrich). Whole blood was used for conducting complete blood counts in an automated CBC-analyzer (Pet care hospital, New Delhi, India).

### Glucose Estimation, Glucose Tolerance Test (GTT), and Lipid Profiling.

The blood glucose levels (5 h fasting) were measured using the Accu-chek blood glucose monitoring system (Roche Diagnostics, Swiss) as per the manufacturer’s instruction. Intraperitoneal GTT was conducted on the final week of PPE treatment. Briefly, mice were kept on a fast for 5 h followed by a d-glucose (1.5 mg/kg) intraperitoneal bolus injection, and measurement of blood glucose at 30, 60, and 120 min was performed after the administration. Plasma cholesterol and triglyceride levels were estimated using an enzymatic assay kit cholesterol E buffer and lab assay triglyceride (Fujifilm Wako pure Chemical Corporation, United States) kit respectively, as per the manufacturer’s protocol.

### Atherosclerotic Plaque Morphometry and Immunostaining

Aortic root embedded in paraffin and sectioned at 8 µm intervals. Six sections at equally spaced intervals were subjected to Hematoxylin and Eosin staining for morphometric analysis including analysis of the total lesional area and necrotic core area. Necrosis was defined as acellular areas characterized by the absence of nuclear staining. Masson’s trichrome staining was performed for the quantification of plaque collagen content.

For immunofluorescence staining, aortic root sections were deparaffinized in xylene followed by antigen retrieval using proteinase K at 37°C for 30 min. The sections were incubated with appropriate primary antibodies: Biotinylated anti-mouse F4/80 (13-4801-82, 1:100, Invitrogen), anti-mouse F4/80 (14-4801-82, 1:100 dilutions, Invitrogen) biotinylated anti-mouse CD3 (36-0031-85, 1:50 dilution, Invitrogen), anti-smooth muscle actin (A2547, 1:100 dilution, Sigma), anti-mouse Mertk (BAF591, 1:100 dilution, R&D Systems), or anti-8-OHdG (BS-1278R, 1:100 dilution, Thermofisher Scientific) for 16 h at 4°C. Subsequently, the sections were incubated with appropriate fluorophore-tagged streptavidin or secondary antibody at indicated dilution for 1 h at room temperature (Streptavidin Alexa flour 647, S32357, 1:1000 dilution, Thermofisher Scientific; Alexa Fluor 594 conjugated goat anti-Mouse IgG, A-11005, 1:1000 dilution, Invitrogen; Alexa Fluor 488 conjugated goat anti-rabbit IgG, A-11008, Invitrogen). Images were captured using EVOS Floid FL-cell imaging system (Invitrogen) and the data were analyzed using ImageJ software.

### Mouse bone marrow-derived macrophage isolation and culture.

10–14 wk old C57BL/6J mice were euthanized and the bone marrows from the femur and tibia were flushed and resuspended in DMEM supplemented with 10% fetal bovine serum and 20% L929-cell culture supernatant. The cells were cultured for 7 days to allow macrophage differentiation with a 50% media change performed on Day 3 of culture.

### TNF and IL-10 ELISA

Plasma levels of TNF and IL-10 were measured using commercially available ELISA kits following the manufacturer’s instructions (Cat# 88-7324-22 and 88-7105-88, Invitrogen). 50 µL of plasma was used per analyte and the final concentrations were calculated following correction for dilution.

### *In vitro* Efferocytosis Assay

Jurkat cells were fluorescently labeled with PKH67 dye (Sigma) as per the manufacturer’s instructions following which the cells were exposed for 5 min to shortwave UV light (UVP). The UV irradiated cells were incubated for 1 h at 37°C to facilitate induction of apoptosis. These fluorescently-labeled apoptotic cells were then added to BMDMs at a ratio of 1:3 (Macrophage: apoptotic cell) and incubated for 2 h. The unengulfed apoptotic cells were washed off and efferocytosis was quantified by fluorescence microscopy.

### TUNEL and *In-situ* Efferocytosis Assay

Apoptotic cells were detected by TUNEL using *In situ* cell death detection kit (Roche). Briefly, deparaffinized lesional sections were incubated with TUNEL reagent as per the manufacturer’s instructions for 30 min at 37°C and counterstained with DAPI to detect the nucleus. Fluorescence microscopic imaging was conducted to detect the TUNEL + nuclei. For *in situ* efferocytosis assay, TUNEL labeling was followed by immunofluorescence labeling with anti-F4/80 antibodies to detect macrophages. Efferocytosis efficiency was estimated as a ratio of TUNEL + nuclei that associate with a macrophage (engulfed) vs. those that are lying free.

### Mertk ELISA

Mertk ELISA was conducted on supernatants of BMDMs or sections of atherosclerotic plaques lysed in protein extraction buffer (300 mM Tris-HCl pH 8.0 and 2% SDS) (Kawashima et al., 2014). Anti-Mertk antibody (AF591, R&D Systems) was used as the capture antibody. 200 µL of culture supernatant or 10 µg equivalent of protein from plaque lysates were loaded per well. Biotinylated anti-Mertk antibody (BAF591, R&D Systems) was used as a detection antibody followed by incubation with streptavidin-HRP and colorimetric development using TMB substrate. A450_nm_ was used to estimate the quantity of soluble Mertk.

### Statistical Analysis

Shapiro–Wilk test was used to test the normal distribution of data. Data that followed Gaussian distribution were analyzed by Student’s two-tailed *t*-test. Non-parametric datasets were analyzed by Mann-Whitney’s *U*-test. ANOVA and multiple comparison tests were conducted when analyzing more than two groups. All data are expressed as mean ± standard error of the mean (SEM).

## Results

### Preparation and Characterization of a Standardized Extract From Pomegranate Peel

Since the bioactive actions of pomegranate peel are mediated by phenolic compounds (Wang et al., 2018), a hydroethanolic extraction procedure was carried out at concentrations ranging from 50–100% to determine the optimal concentration that provides the maximal yield and phenolic content. 60% hydroethanolic extraction exhibited a maximal total yield of 43.10 ± 0.14% (Table 1) along with the highest content of phenolic compounds and flavonoids (Table 2). Furthermore, UPLC-based analysis demonstrated that the 60% hydroethanolic extract contained the highest levels of bioactive punicalagin A and punicalagin B (Table 3). Thus, a standardized extraction procedure using 60% hydroethanolic was used further in this study.

**TABLE 1:** Extraction yield of pomegranate peel: The data are derived from 3 independent extractions and are represented as Mean ± SD.

sample	Whole pomegranate peel					
	50%	60%	70%	80%	90%	100%
Extraction yield (%)	22.0 ± 0.04	43.10 ± 0.14	40.50 ± 0.16	42.80 ± 0.04	38.71 ± 0.09	24.40 ± 0.16

**TABLE 2:** Total phenolic and flavonoid content of pomegranate peel extract. The data are derived from 3 independent extractions and are represented as Mean ± SD. TPC, total phenolic content; TFC, total flavonoid content.

Sample	Whole pomegranate peel					
	50%	60%	70%	80%	90%	100%
TPC (mg GAE/g)	114.01 ± 08	125.24 ± 0.11	123.11 ± 0.10	121.68 ± 0.25	97.18 ± 0.19	96.33 ± 0.26
TFC (mg RU/g)	34.21 ± 0.12	57.96 ± 0.15	50.12 ± 0.24	43.61 ± 0.18	41.25 ± 0.18	51.3 ± 0.07

**TABLE 3:** UPLC profiling of pomegranate peel for different phenolic compounds under different hydroethanolic concentration. The data are derived from 3 independent extractions and are represented as Mean ± SD. NQ, not quantifiable.

Hydroethanolic extracts (%)	Phenolic compounds (µg/10 mg)					
	Gallic acid	Chlorogenic acid	*p*-Coumaric acid	Caffeic acid	Punicalagin A	Punicalagin B
50	0.7 ± 0.02	9.1 ± 0.05	5.5 ± 0.01	2.3 ± 0.02	178.83 ± 0.01	115.07 ± 0.04
60	1.2 ± 0.04	19.6 ± 0.03	11.3 ± 0.02	5.5 ± 0.03	630.88 ± 0.03	494.81 ± 0.05
70	NQ	8.4 ± 0.01	5.0 ± 0.04	1.9 ± 0.05	248.4 ± 0.02	255.6 ± 0.02
80	0.4 ± 0.01	9.8 ± 0.02	10.5 ± 0.01	1.9 ± 0.01	558.80 ± 0.01	321.83 ± 0.03
90	0.8 ± 0.03	10.5 ± 0.04	9.4 ± 0.02	2.0 ± 0.04	555.08 ± 0.04	294.50 ± 0.01
100	0.9 ± 0.01	9.9 ± 0.02	9.1 ± 0.03	1.9 ± 0.05	568.83 ± 0.03	302.20 ± 0.02

### Pomegranate Peel Extract Improves Metabolic Profile of Western Diet Fed *Apoe* ^*-/-*^ Mice

**FIGURE 1:**
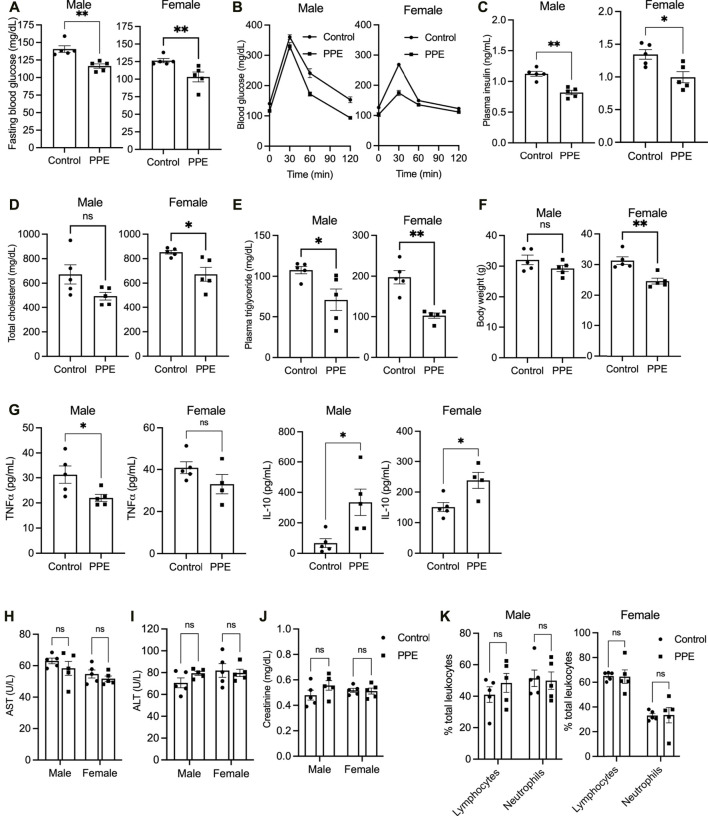
10 male and 10 female *Apoe* ^*-/-*^ mice were randomized into two groups of 5 mice each, one receiving vehicle and another receiving pomegranate peel extract (200 mg/kg body weight) per oral daily during the entire duration of 12 weeks when they were fed western-type diet. At the end of 12 weeks of treatment, the following parameters were measured **(A)** fasting blood glucose **(B)** intraperitoneal glucose tolerance test **(C)** plasma insulin **(D)** plasma total cholesterol **(E)** plasma triglyceride **(F)** body weight **(G)** plasma TNF and IL-10 levels by ELISA **(H)** plasma AST levels **(I)** plasma ALT levels **(J)** plasma creatinine, and **(K)** differential leukocyte count. All data were tested for statistical significance using Mann–Whitney test. ns, no significant difference; *, p < 0.05; **, p < 0.01. TNF and IL-10 ELISA were conducted on 5 control and 4 PPE-treated female mice due to insufficient sample volume in one PPE-treated female mouse.

Eight-week-old male and female *Apoe* ^*-/-*^ mice were fed a western type (high-fat high-cholesterol) diet ad libitum. The mice were randomized into two groups, one receiving a vehicle while the other group was administered pomegranate peel extract (PPE, 200 mg/kg) daily *via* oral route for 12 weeks. This dose was chosen based on our previous study wherein administration of mice with 200 mg/kg PPE led to maximal cardioprotective effect in an isoproterenol-induced myocardial infarction model (Gupta et al., 2015). Mice that were given PPE demonstrated significantly lower levels of blood glucose (Figure 1A) and showed enhanced glucose tolerance (Figure 1B). Interestingly, the PPE-fed mice had lower plasma insulin levels (Figure 1C) suggesting that the lowering of fasting glucose in the PPE-fed mice could be mediated by improved insulin sensitivity in peripheral tissues. Additionally, PPE-fed mice demonstrated significantly lower levels of plasma total cholesterol (Figure 1D) and triglycerides (Figure 1E). Interestingly, the body weight was similar between the control and PPE-fed group in the male mice, but the PPE-fed female mice demonstrated decreased weight gain (Figure 1F) despite eating a similar amount of food (3.1 ± 0.4 g/day *vs* 3.3 ± 0.3 g/day). Additionally, systemic inflammation was lower in the PPE-fed mice as demonstrated by decreased plasma level of pro-inflammatory cytokine TNF and increased expression of anti-inflammatory cytokine IL-10 (Figure 1G). Most importantly, the levels of aspartate aminotransferase (AST), alanine aminotransferase (ALT), and creatinine were similar between the control and PPE-fed mice (Figures 1H–J) demonstrating that chronic administration of PPE did not result in hepatotoxicity or nephrotoxicity. Additionally, the total and differential leukocyte counts were similar between the control and PPE-fed mice (Figure 1K). In summary, these data suggest that PPE improves metabolic and lipid profiles in the western diet-fed male and female *Apoe* ^*-/-*^ mice.

### Pomegranate Peel Extract Decreases Plaque Necrosis and Increases Collagen Deposition in Advanced Atherosclerotic Lesions

**FIGURE 2:**
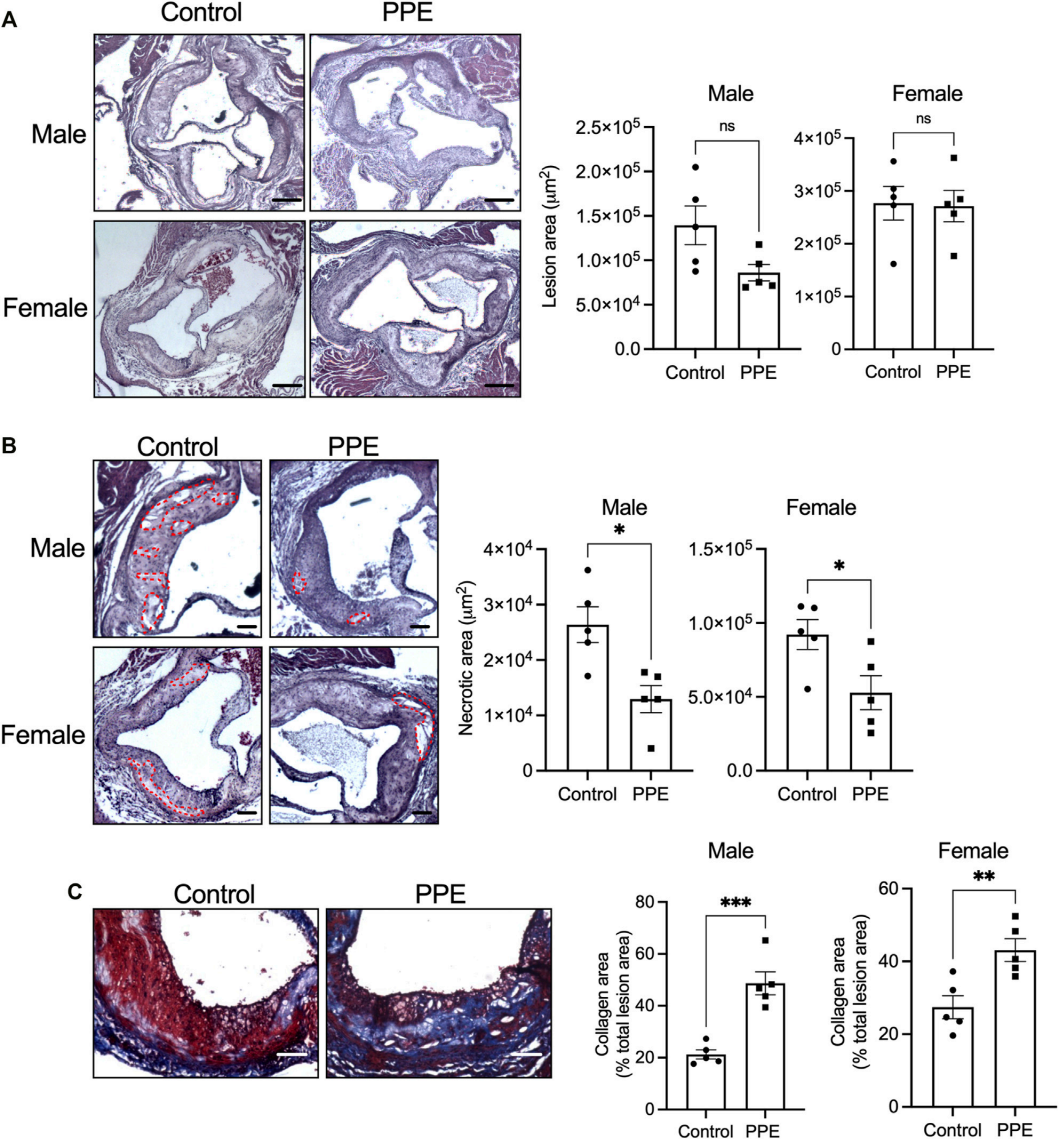
H&E-stained sections of aortic root from male and female mice treated with PPE or vehicle were used for quantification of **(A)** total lesion area and **(B)** necrotic area. Regions of necrosis are highlighted using red dotted lines. Bar, 200 µm **(A),** and 100 µm **(B) (C)** Aortic root sections were stained with Masson trichrome stain for quantification of collagen content in the intimal region of the plaque. Bar, 50 μm *n* = 5 mice per group per sex. Statistical significance was analyzed using Mann–Whitney test. ns, no significant difference; *, p < 0.05; **, p < 0.01; ***, p < 0.001.

**FIGURE 3:**
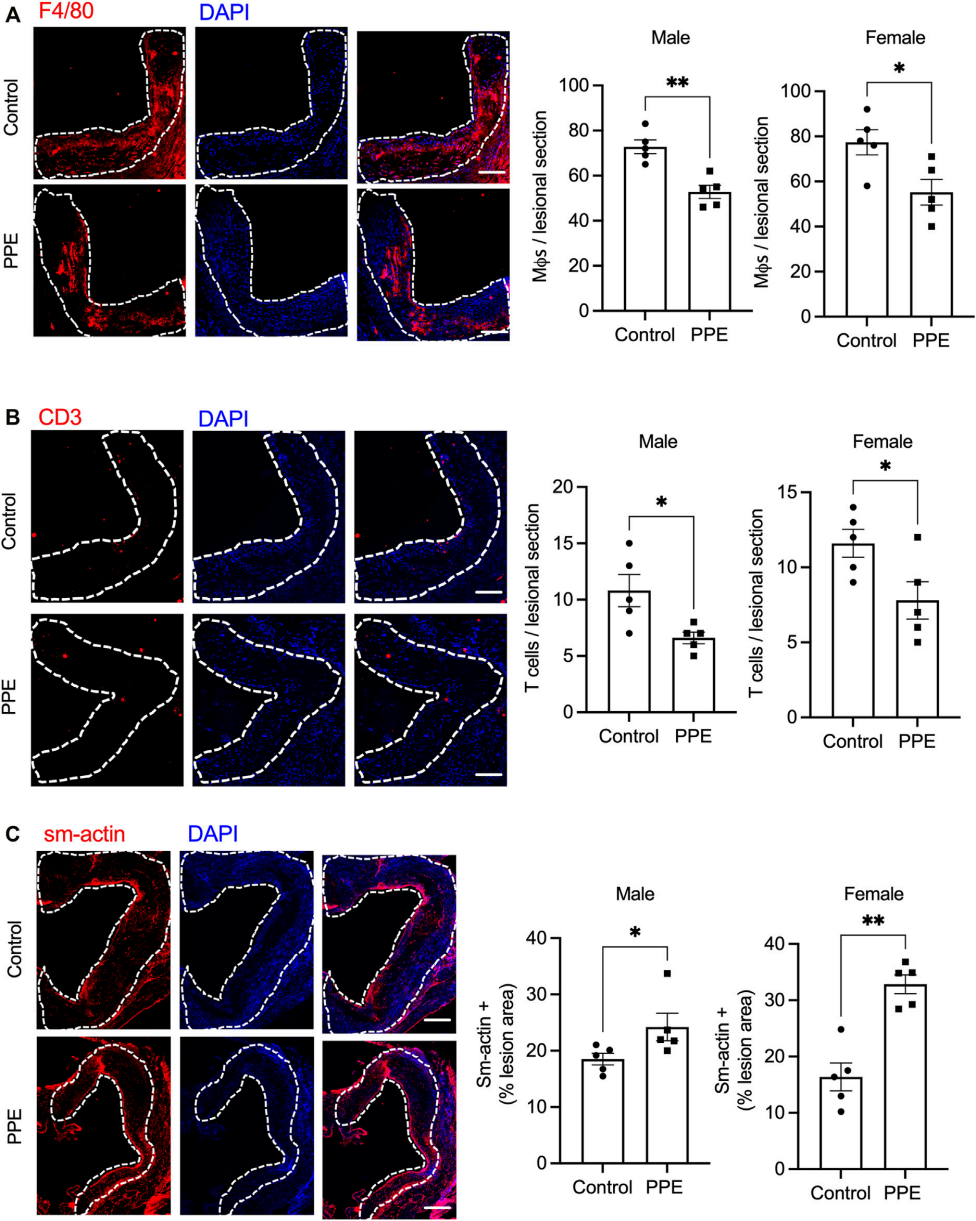
Aortic root sections from male and female mice treated with PPE or vehicle were immunostained with **(A)** anti-F4/80 antibody **(B)** anti-CD3 antibody, or **(C)** anti-smooth muscle actin antibody followed by fluorescence microscopy for imaging. The number of F4/80+ (macrophages), sm-actin + (smooth muscle cells), and CD3+ (T-cells) cells in control or PPE-treated groups were quantified using ImageJ. The dotted lines demarcate the intimal region. Bar, 50 μm *n* = 5 mice per group per sex. Analysis of statistical significance was conducted by Mann-Whitney test. *, p < 0.05; **, p < 0.01.

We next examined the effect of PPE on atherosclerotic plaque parameters. Despite an improvement in metabolic profile, PPE-fed mice had similar total atherosclerotic lesion area as compared with control mice (Figure 2A). However, plaque necrosis, which is a critical parameter associated with plaque rupture and clinical manifestations of myocardial infarction and stroke in humans (Finn et al., 2010), was significantly decreased in the PPE-fed mice (Figure 2B). Additionally, PPE-fed mice demonstrated increased lesional collagen content (Figure 2C), which in humans is associated with plaque stability and improved cardiovascular outcomes. (Finn et al., 2010; Virmani et al., 2000). Next, we analyzed the cellular heterogeneity of the plaques by immunophenotyping. Atherosclerotic lesions of PPE-fed mice had significantly lower numbers of F4/80 + macrophages (Figure 3A) and CD3^+^ T cells (Figure 3B), and increased numbers of smooth muscle cells (Figure 3C). Since, the accumulation of macrophages and T-cells in the plaque are associated with adverse atherothrombotic complications (Tabas and Lichtman, 2017), these data demonstrate that although PPE treatment did not change the total lesion size, it led to a matrix and cellular remodeling of the plaque which is consistent with a “clinically favorable” stable-plaque phenotype.

### Pomegranate Peel Extract Mediated Reduction in Plaque Necrosis is Associated with Decreased Mertk Cleavage and Improved Lesional Efferocytosis.

**FIGURE 4:**
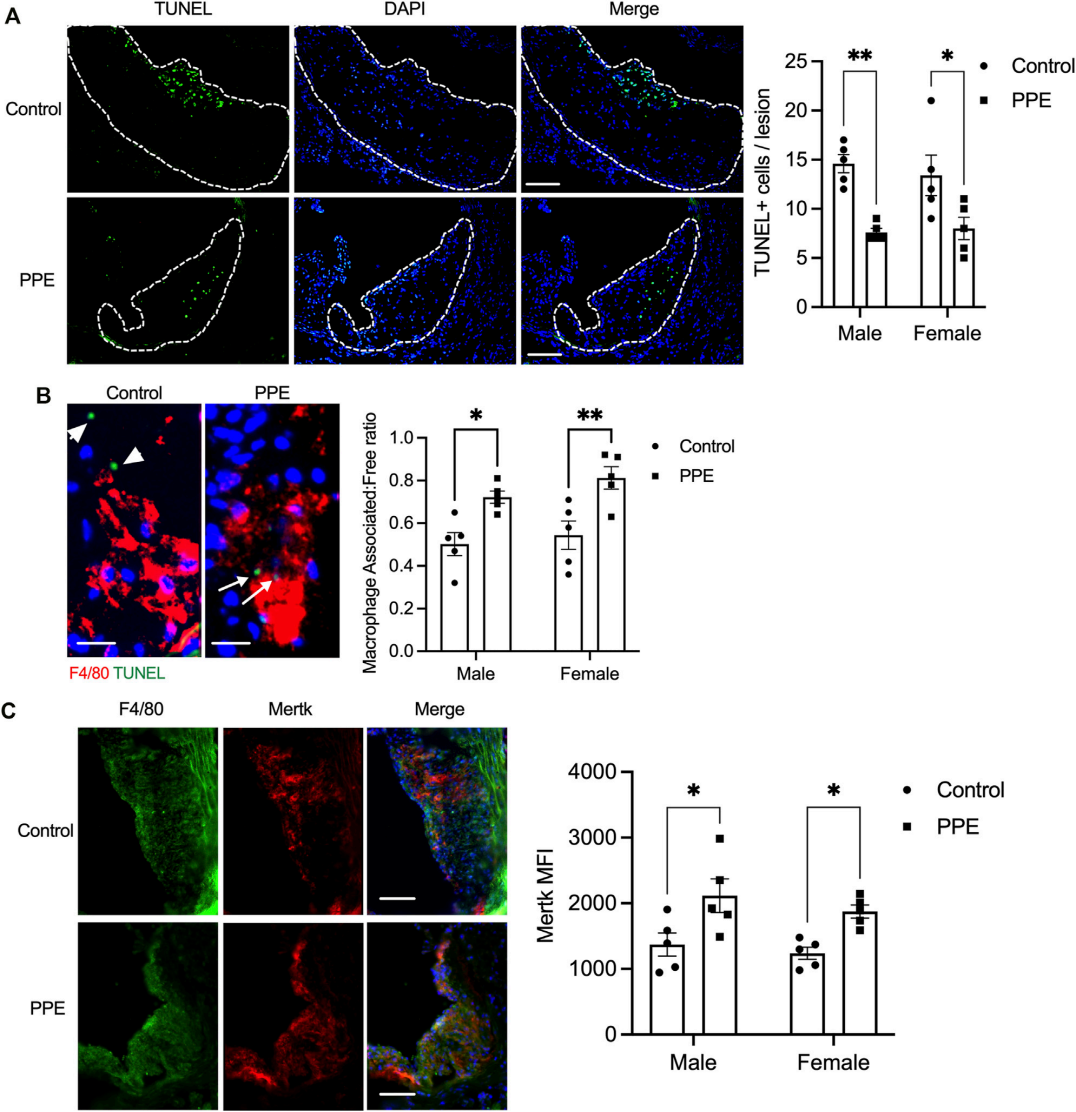
**(A)** TUNEL assay was conducted on aortic root sections from male and female mice treated with PPE or vehicle to determine the number of dead cells in the atherosclerotic plaque. Bar, 50 μm **(B)** In-situ efferocytosis assay was conducted by immunostaining macrophages with anti-F4/80 antibody (red) and dead cells using TUNEL reagent (green). The number of TUNEL + nuclei that were either associated with an F4/80 + macrophage (white arrow) or were lying free (white arrowheads) was quantified. The associated: free ratio was used as a measure of lesional macrophage efferocytosis efficiency. Bar, 10 µm **(C)** Immunostaining of aortic root sections with anti-F4/80 and anti-Mertk antibody. The mean fluorescence intensity of the staining was quantified in ImageJ by drawing an ROI around the F4/80 + intimal region of the plaque. Bar, 50 µm. Non-parametric Mann-Whitney U test was conducted to determine statistical significance. *, p < 0.05; **, p < 0.01.

Expansion of the necrotic core in the atherosclerotic plaque is facilitated by a combination of increased lesional cell death and defective efferocytosis (phagocytic clearance of apoptotic cells) (Kasikara et al., 2018; Dhawan et al., 2021). Since we observed decreased plaque necrosis in the PPE-fed mice, we analyzed whether lesional efferocytosis or lesional death was altered. Compared with the control mice, PPE-fed mice had significantly lower numbers of TUNEL + cells in the intimal region of the plaque (Figure 4A). Next, we conducted an *in-situ* efferocytosis assay (Thorp et al., 2008) to quantify lesional efferocytosis efficiency as a ratio of TUNEL + apoptotic cells that are either associated with an F4/80 + macrophage or lying free (Associated: free ratio). Interestingly, the PPE-treated mice demonstrated a significant increase in lesional efferocytosis efficiency (Figure 4B).

**FIGURE 5:**
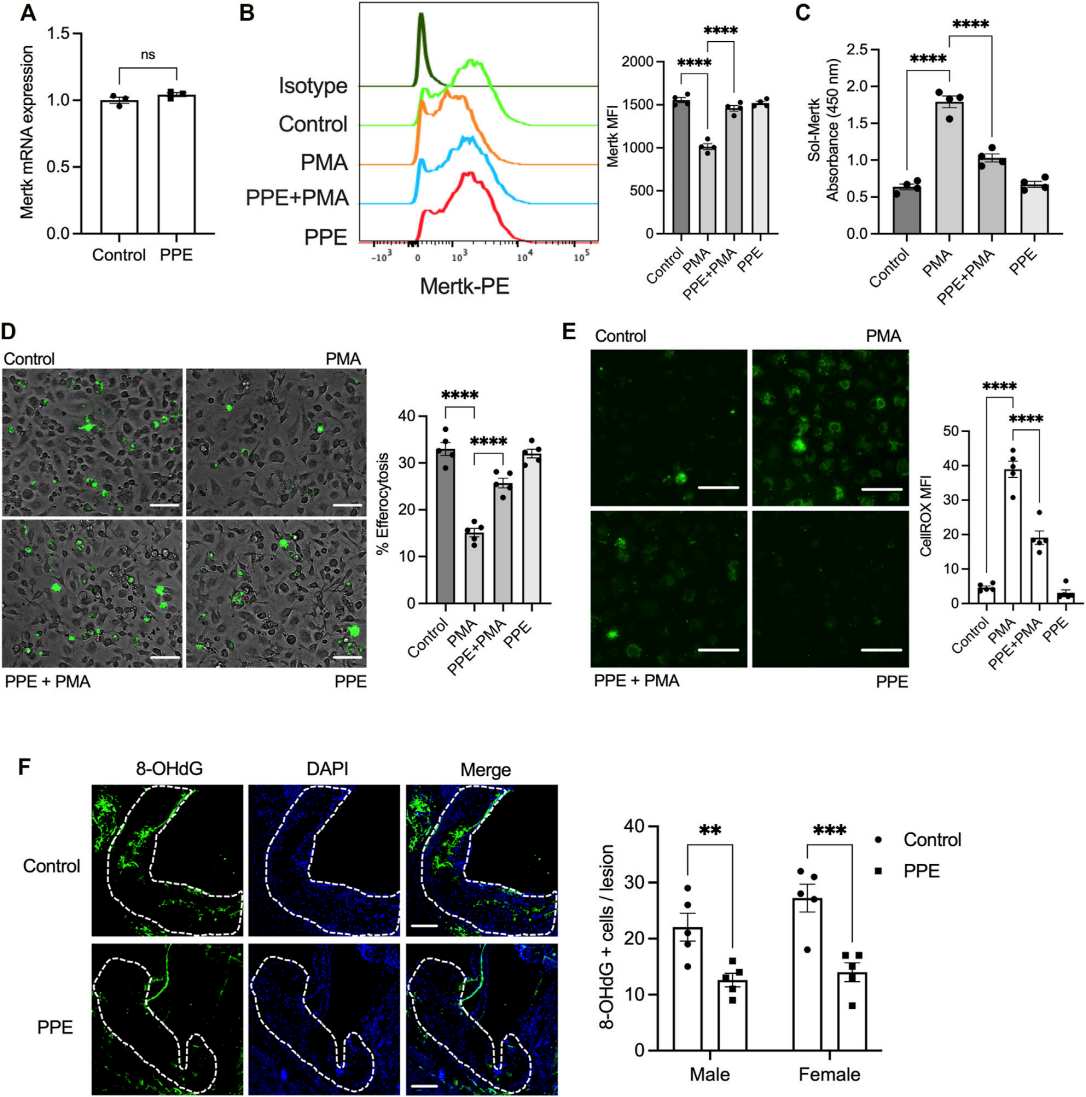
**(A)** qPCR-based analysis of Mertk mRNA expression in BMDMs treated with vehicle or 50 μg/ml PPE for 16 h *n* = 3 **(B)** Flow-cytometry based analysis of cell surface Mertk expression on BMDMs incubated with 50 μg/ml PPE for 16 h followed by treatment with 50 nM PMA for 2 h. The bar graph represents means fluorescence intensity derived from the histogram. *n* = 4 **(C)** The levels of soluble Mertk (Sol-Mertk) in the supernatants of BMDMs treated with PMA in the absence or presence of PPE (50 μg/ml) were analyzed by a sandwich ELISA. The bar graph represents the absorbance values measured at 450 nm *n* = 4 **(D)** Efferocytosis assay was conducted by incubating fluorescently labeled apoptotic cells (green) with BMDMs treated with PMA (50 nM) in the absence or presence of PPE (50 μg/ml). Bar, 20 μm *n* = 4 **(E)** Fluorescence microscopy-based analysis of cytosolic ROS by CellROX staining in BMDMs incubated with vehicle or PPE (50 μg/ml) for 16 h followed by treatment with 50 nM PMA for 2 h. Bar, 20 μm *n* = 4 **(F)** Aortic root sections of control or PPE-fed male and female *Apoe* ^*-/-*^ mice (*n* = 5 mice per group per sex) were immunostained with anti-8-OHdG to assay lesional ROS-mediated damage. The white dotted line demarcates the intimal region of the plaque. Data were tested for statistical significance using a two-tailed Student’s t-test **(A)**, one-way ANOVA with Sidak’s multiple comparisons test **(B–E)**, or Mann–Whitney test **(F)**. ns, no significant difference; *, p < 0.05; **, p < 0.01; ***, p < 0.001; ****, p < 0.0001.

One of the key mechanisms by which efferocytosis is impaired in advanced atherosclerotic plaque involves enzymatic cleavage of the efferocytosis receptor(s) by the ADAM family of sheddases (Thorp et al., 2011; Cai et al., 2017). Since PPE-treated mice had a higher lesional efferocytosis efficiency, we tested whether PPE was able to prevent efferocytosis receptor shedding and retain high-level expression of efferocytosis receptors on lesional macrophages. Indeed, we observed that the expression levels of Mertk, one of the key macrophage efferocytosis receptor, was significantly higher in the lesional macrophages of the PPE-treated mice (Figure 4C). The increased expression levels of Mertk could be mediated either by transcriptional upregulation of Mertk or *via* inhibition of ADAM17-mediated cleavage of Mertk. Mertk mRNA levels were not affected by PPE treatment in BMDMs (Figure 5A) suggesting that PPE regulates Mertk expression by a post-transcriptional mechanism. Next, we tested whether PPE affects Mertk cleavage in macrophages. Cleavage of Mertk results in the release of the ectodomain of Mertk (soluble-Mertk, Sol-Mer) into the adjacent extracellular space (Thorp et al., 2011; Sather et al., 2007). Interestingly, PPE was able to block PMA-mediated cleavage of Mertk in murine BMDMs *in vitro* as demonstrated by the increased levels of cell surface Mertk expression (Figure 5B) and decreased levels of Sol-Mertk in the supernatant of macrophages treated with PPE (Figure 5C). Consistent with the ability of PPE to block Mertk cleavage, PPE treatment reversed the defect in macrophage efferocytosis mediated by PMA (Figure 5D). PMA mediates cleavage of Mertk *via* ROS-dependent activation of ADAM17. In this context, we asked whether PPE suppresses Mertk cleavage *via* its antioxidant activity. Indeed, PPE treatment led to significant suppression of PMA-induced generation of ROS in macrophages (Figure 5E). Consistent with these data, we observed that 8-OHdG, a marker of oxidative stress-mediated cellular damage. (Valavanidis et al., 2009; Wang et al., 2014), was significantly lower in the atherosclerotic plaques of PPE-treated mice (Figure 5F). These data taken together suggest that PPE improves macrophage efferocytosis efficiency in advanced atherosclerotic lesions *via* suppressing the cleavage of Mertk through its antioxidant capacity.

## Discussion

Several lines of evidence demonstrate the potent vasculoprotective and cardioprotective effects of pomegranate juice and extracts on early atherosclerotic lesion parameters in animal models of disease as well as in patients with coronary artery disease (Aviram et al., 2000; Aviram et al., 2004; Wang et al., 2018). In this study, we extend these findings to demonstrate that a standardized hydroethanolic extract of pomegranate peel that is rich in polyphenols including punicalagin decreases advanced atherosclerotic plaque necrosis presumably by promoting lesional macrophage efferocytosis *via* suppression of Mertk cleavage.

The myriad beneficial effects of natural antioxidants on health and well-being have piqued the interest of consumers in plant-based products. Thus, the extraction of bioactive compounds from food and agricultural waste represents an attractive strategy to enhance sustainable agricultural practices. Indeed, several foods and agricultural waste products have been tested for the efficient extraction of polyphenolic compounds. In this context, pomegranate peel represents about 50% of the net weight of the fruit and is usually discarded as a waste product. However, it is rich in phenolic compounds and antioxidants such as epicatechin, gallic acid, and punicalagin which are a potent source of cardioprotective bioactive molecules (Li et al., 2006; Wang et al., 2018).

Atherosclerosis and its associated complications are driven by a combination of factors including dyslipidemia, insulin resistance, and chronic non-resolving inflammation (Libby et al., 2019). Indeed, the treatment strategy for atherosclerosis involves a multipronged approach to lower known risk factors including lifestyle modifications and lowering plasma cholesterol and triglycerides, management of hypertension, and establishment of euglycemia (Arnett et al., 20192019). Previous studies have demonstrated that pomegranate juice and pomegranate peel extract promote the establishment of a favorable lipid and glucose profile in patients with coronary heart disease as well as in pre-clinical animal models (Aviram et al., 2000; Aviram et al., 2004; Wang et al., 2018). The mechanism involves both suppressions of triglyceride biosynthesis and increased hepatic and macrophage-mediated metabolism of lipids such as *via* suppression of DGAT-1 activity (Rosenblat and Aviram, 2011), enhanced uptake of LDL by macrophages (Atrahimovich et al., 2016), increased cholesterol efflux (Zhao et al., 2016), and increased expression of Cyp7a1 and Cyp7b1 thereby regulating cholesterol excretion (Yang et al., 2018). Many of these studies were conducted for a short duration ranging from 1–6 weeks and thus whether the beneficial effects of pomegranate extract persist for a longer duration to promote cardiovascular health was unknown. Interestingly, our study demonstrates that the lowering of plasma cholesterol, triglyceride, and glucose by pomegranate peel extract is sustained over 12 weeks. Most importantly, our data also demonstrates that PPE-fed mice had lower lesional and systemic inflammation which may promote the favorable remodeling of atherosclerotic plaque.

In a genetic model where the mice are resistant to cleavage of the key efferocytosis receptor Mertk, the atherosclerotic plaques demonstrated enhanced lesional efferocytosis capacity, decreased necrotic core area, lower lesional inflammation, and increased plaque collagen content (Cai et al., 2017). These data are similar to our observation in the PPE-fed mice that showed enhanced lesional Mertk expression levels which were associated with increased efferocytosis efficiency, decreased lesional necrosis, and enhanced collagen deposition. Interestingly, ADAM17 cleaves the ectodomain of Mertk to yield soluble Mertk (Sol-Mer). (Sather et al., 2007; Thorp et al., 2011) which is one of the key mechanisms that lead to decreased lesional macrophage efferocytosis in advanced atherosclerotic plaques (Garbin et al., 2012; Cai et al., 2017). ADAM17 is activated by a ROS- p38-MAPK pathway (Thorp et al., 2011). In this context, PPE-fed mice demonstrated lower lesional ROS suggesting that PPE-mediated antioxidant activity might result in suppression of ADAM17 activation and decreased cleavage of macrophage Mertk.

Expansion of the necrotic core in advanced atherosclerotic plaque is mediated by defective efferocytosis in the background of increased lesional cell death. Therefore, correction of defective efferocytosis in the lesion may promote inflammation resolution and stall atherosclerosis progression. Since pomegranate polyphenols have been reported to increase macrophage apoptosis (Rom et al., 2016), our data suggest that pomegranate products may lower atherosclerosis progression *via* dual action of increasing lesional cell death coupled to efficient clearance of dying cells. To our knowledge, this is the first report of a natural product extract that can suppress Mertk cleavage on macrophages. Since the cleavage of Mertk is associated with impaired inflammation resolution in several pathological states. (Wu et al., 2011; Zizzo et al., 2013; DeBerge et al., 2017; Rovati et al., 2021), our findings suggest that pomegranate peel extract may be beneficial under these conditions and warrants further investigation.

## Data Availability

The original contributions presented in the study are included in the article/supplementary material; further inquiries can be directed to the corresponding author.

## References

[B1] Arnett D. K. Blumenthal R. S. Albert M. A. Buroker A. B. Goldberger Z. D. Hahn E. J. (20192019). 2019 ACC/AHA Guideline on the Primary Prevention of Cardiovascular Disease: Executive Summary: A Report of the American College of Cardiology/American Heart Association Task Force on Clinical Practice Guidelines. J. Am. Coll. Cardiol. 74 (10), 1376–1414. 10.1016/j.jacc.2019.03.009 PMC8344373 30894319

[B2] Atrahimovich D. Khatib S. Sela S. Vaya J. Samson A. O. (2016). Punicalagin Induces Serum Low-Density Lipoprotein Influx to Macrophages. Oxid. Med. Cell Longev. 2016, 7124251. 10.1155/2016/7124251 27516832 PMC4969581

[B3] Aviram M. Dornfeld L. Rosenblat M. Volkova N. Kaplan M. Coleman R. (2000). Pomegranate Juice Consumption Reduces Oxidative Stress, Atherogenic Modifications to LDL, and Platelet Aggregation: Studies in Humans and in Atherosclerotic Apolipoprotein E-Deficient Mice. Am. J. Clin. Nutr. 71 (5), 1062–1076. 10.1093/ajcn/71.5.1062 10799367

[B4] Aviram M. Rosenblat M. Gaitini D. Nitecki S. Hoffman A. Dornfeld L. (2004). Pomegranate Juice Consumption for 3 Years by Patients with Carotid Artery Stenosis Reduces Common Carotid Intima-Media Thickness, Blood Pressure and LDL Oxidation. Clin. Nutr. 23 (3), 423–433. 10.1016/j.clnu.2003.10.002 15158307

[B5] Bhatt S. Kumari N. Abhishek V. Gupta M. (2021). Elucidating the Role of Amaranth Flour in Formulation of Gluten Free Black Rice Muffins and its Premix: Nutritional, Physico-Chemical and Textural Characteristics. Food Meas. 15 (1), 675–685. 10.1007/s11694-020-00675-y

[B6] Bhatt S. Singh B. Gupta M. (2020). Antioxidant and Prebiotic Potential of Murraya Koenigii and Brassica oleracea Var. Botrytis Leaves as Food Ingredient. J. Agric. Food Res. 2, 100069. 10.1016/j.jafr.2020.100069

[B7] Cai B. Thorp E. B. Doran A. C. Sansbury B. E. Daemen M. J. Dorweiler B. (2017). MerTK Receptor Cleavage Promotes Plaque Necrosis and Defective Resolution in Atherosclerosis. J. Clin. Invest 127 (2), 564–568. 10.1172/JCI90520 28067670 PMC5272169

[B8] DeBerge M. Yeap X. Y. Dehn S. Zhang S. Grigoryeva L. Misener S. (2017). MerTK Cleavage on Resident Cardiac Macrophages Compromises Repair after Myocardial Ischemia Reperfusion Injury. Circ. Res. 121 (8), 930–940. 10.1161/CIRCRESAHA.117.311327 28851810 PMC5623080

[B9] Dhawan U. K. Singhal A. Subramanian M. (2021). Dead Cell and Debris Clearance in the Atherosclerotic Plaque: Mechanisms and Therapeutic Opportunities to Promote Inflammation Resolution. Pharmacol. Res. 170, 105699. 10.1016/j.phrs.2021.105699 34087352

[B10] Finn A. V. Nakano M. Narula J. Kolodgie F. D. Virmani R. (2010). Concept of Vulnerable/Unstable Plaque. Arterioscler. Thromb. Vasc. Biol. 30 (7), 1282–1292. 10.1161/ATVBAHA.108.179739 20554950

[B11] Garbin U. Baggio E. Stranieri C. Pasini A. Manfro S. Mozzini C. (2012). Expansion of Necrotic Core and Shedding of Mertk Receptor in Human Carotid Plaques: a Role for Oxidized Polyunsaturated Fatty Acids? Cardiovasc Res. 97 (1), 125–133. 10.1093/cvr/cvs301 22997156

[B12] Gupta M. Sharma P. Mazumder A. G. Patial V. Singh D. (2015). Dwindling of Cardio Damaging Effect of Isoproterenol by Punica granatum L. Peel Extract Involve Activation of Nitric Oxide-Mediated Nrf2/ARE Signaling Pathway and Apoptosis Inhibition. Nitric Oxide 50, 105–113. 10.1016/j.niox.2015.09.002 26363155

[B13] Jarr K.-U. Nakamoto R. Doan B. H. Kojima Y. Weissman I. L. Advani R. H. (2021). Effect of CD47 Blockade on Vascular Inflammation. N. Engl. J. Med. 384 (4), 382–383. 10.1056/nejmc2029834 33503349 PMC8175009

[B14] Kasikara C. Doran A. C. Cai B. Tabas I. (2018). The Role of Non-resolving Inflammation in Atherosclerosis. J. Clin. Invest 128 (7), 2713–2723. 10.1172/JCI97950 30108191 PMC6025992

[B15] Kawashima Y. Kodera Y. Singh A. Matsumoto M. Matsumoto H. (2014). Efficient Extraction of Proteins from Formalin-Fixed Paraffin-Embedded Tissues Requires Higher Concentration of Tris(hydroxymethyl)aminomethane. Clin. Proteomics 11 (1), 4. 10.1186/1559-0275-11-4 24484752 PMC3922997

[B16] Kirichenko T. V. Sukhorukov V. N. Markin A. M. Nikiforov N. G. Liu P. Y. Sobenin I. A. (2020). Medicinal Plants as a Potential and Successful Treatment Option in the Context of Atherosclerosis. Front. Pharmacol. 11 (403), 403. 10.3389/fphar.2020.00403 32322201 PMC7156611

[B17] Kojima Y. Volkmer J. P. McKenna K. Civelek M. Lusis A. J. Miller C. L. (2016). CD47-blocking Antibodies Restore Phagocytosis and Prevent Atherosclerosis. Nature 536 (7614), 86–90. 10.1038/nature18935 27437576 PMC4980260

[B18] Li Y. Guo C. Yang J. Wei J. Xu J. Cheng S. (2006). Evaluation of Antioxidant Properties of Pomegranate Peel Extract in Comparison with Pomegranate Pulp Extract. Food Chem. 96 (2), 254–260. 10.1016/j.foodchem.2005.02.033

[B19] Libby P. Buring J. E. Badimon L. Hansson G. K. Deanfield J. Bittencourt M. S. (2019). Atherosclerosis. Nat. Rev. Dis. Prim. 5 (1), 56. 10.1038/s41572-019-0106-z 31420554

[B20] Rodriguez F. Maron D. J. Knowles J. W. Virani S. S. Lin S. Heidenreich P. A. (2017). Association between Intensity of Statin Therapy and Mortality in Patients with Atherosclerotic Cardiovascular Disease. JAMA Cardiol. 2 (1), 47–54. 10.1001/jamacardio.2016.4052 27829091

[B21] Rom O. Volkova N. Nandi S. Jelinek R. Aviram M. (2016). Pomegranate Juice Polyphenols Induce Macrophage Death via Apoptosis as Opposed to Necrosis Induced by Free Radical Generation: A Central Role for Oxidative Stress. J. Cardiovasc Pharmacol. 68 (2), 106–114. 10.1097/FJC.0000000000000391 27010808

[B22] Rosenblat M. Aviram M. (2011). Pomegranate Juice Protects Macrophages from Triglyceride Accumulation: Inhibitory Effect on DGAT1 Activity and on Triglyceride Biosynthesis. Ann. Nutr. Metab. 58 (1), 1–9. 10.1159/000323096 21212659

[B23] Rovati L. Kaneko N. Pedica F. Monno A. Maehara T. Perugino C. (2021). Mer Tyrosine Kinase as a Possible Link between Resolution of Inflammation and Tissue Fibrosis in IgG4-Related Disease. Rheumatol. Oxf. 60 (10), 4929–4941. 10.1093/rheumatology/keab096 PMC8487308 33512463

[B24] Sather S. Kenyon K. D. Lefkowitz J. B. Liang X. Varnum B. C. Henson P. M. (2007). A Soluble Form of the Mer Receptor Tyrosine Kinase Inhibits Macrophage Clearance of Apoptotic Cells and Platelet Aggregation. Blood 109 (3), 1026–1033. 10.1182/blood-2006-05-021634 17047157 PMC1785151

[B25] Tabas I. Lichtman A. H. (2017). Monocyte-Macrophages and T Cells in Atherosclerosis. Immunity 47 (4), 621–634. 10.1016/j.immuni.2017.09.008 29045897 PMC5747297

[B26] Thorp E. Cui D. Schrijvers D. M. Kuriakose G. Tabas I. (2008). Mertk Receptor Mutation Reduces Efferocytosis Efficiency and Promotes Apoptotic Cell Accumulation and Plaque Necrosis in Atherosclerotic Lesions of Apoe-/- Mice. Arterioscler. Thromb. Vasc. Biol. 28 (8), 1421–1428. 10.1161/ATVBAHA.108.167197 18451332 PMC2575060

[B27] Thorp E. Vaisar T. Subramanian M. Mautner L. Blobel C. Tabas I. (2011). Shedding of the Mer Tyrosine Kinase Receptor Is Mediated by ADAM17 Protein through a Pathway Involving Reactive Oxygen Species, Protein Kinase Cδ, and P38 Mitogen-Activated Protein Kinase (MAPK). J. Biol. Chem. 286 (38), 33335–33344. 10.1074/jbc.M111.263020 21828049 PMC3190938

[B28] Valavanidis A. Vlachogianni T. Fiotakis C. (2009). 8-hydroxy-2' -deoxyguanosine (8-OHdG): A Critical Biomarker of Oxidative Stress and Carcinogenesis. J. Environ. Sci. Health C Environ. Carcinog. Ecotoxicol. Rev. 27 (2), 120–139. 10.1080/10590500902885684 19412858

[B29] Virani S. S. Alonso A. Aparicio H. J. Benjamin E. J. Bittencourt M. S. Callaway C. W. (2021). Heart Disease and Stroke Statistics-2021 Update: A Report from the American Heart Association. Circulation 143 (8), e254–e743. 10.1161/CIR.0000000000000950 33501848 PMC13036842

[B30] Virmani R. Kolodgie F. D. Burke A. P. Farb A. Schwartz S. M. (2000). Lessons from Sudden Coronary Death: a Comprehensive Morphological Classification Scheme for Atherosclerotic Lesions. Arterioscler. Thromb. Vasc. Biol. 20 (5), 1262–1275. 10.1161/01.atv.20.5.1262 10807742

[B31] Wang D. Özen C. Abu-Reidah I. M. Chigurupati S. Patra J. K. Horbanczuk J. O. (2018). Vasculoprotective Effects of Pomegranate (Punica granatum L.). Front. Pharmacol. 9 (544), 544. 10.3389/fphar.2018.00544 29881352 PMC5977444

[B32] Wang Y. Wang G. Z. Rabinovitch P. S. Tabas I. (2014). Macrophage Mitochondrial Oxidative Stress Promotes Atherosclerosis and Nuclear Factor-Κb-Mediated Inflammation in Macrophages. Circ. Res. 114 (3), 421–433. 10.1161/CIRCRESAHA.114.302153 24297735 PMC3946745

[B33] Wu J. Ekman C. Jönsen A. Sturfelt G. Bengtsson A. A. Gottsäter A. (2011). Increased Plasma Levels of the Soluble Mer Tyrosine Kinase Receptor in Systemic Lupus Erythematosus Relate to Disease Activity and Nephritis. Arthritis Res. Ther. 13 (2), R62. 10.1186/ar3316 21496228 PMC3132057

[B34] Yang J. Zhang S. Henning S. M. Lee R. Hsu M. Grojean E. (2018). Cholesterol-lowering Effects of Dietary Pomegranate Extract and Inulin in Mice Fed an Obesogenic Diet. J. Nutr. Biochem. 52, 62–69. 10.1016/j.jnutbio.2017.10.003 29172112

[B35] Yurdagul A. Doran A. C. Cai B. Fredman G. Tabas I. A. (2018). Mechanisms and Consequences of Defective Efferocytosis in Atherosclerosis. Front. Cardiovasc Med. 4 (86), 86. 10.3389/fcvm.2017.00086 29379788 PMC5770804

[B36] Zhao S. Li J. Wang L. Wu X. (2016). Pomegranate Peel Polyphenols Inhibit Lipid Accumulation and Enhance Cholesterol Efflux in raw264.7 Macrophages. Food Funct. 7 (7), 3201–3210. 10.1039/c6fo00347h 27334099

[B37] Zizzo G. Guerrieri J. Dittman L. M. Merrill J. T. Cohen P. L. (2013). Circulating Levels of Soluble MER in Lupus Reflect M2c Activation of Monocytes/macrophages, Autoantibody Specificities and Disease Activity. Arthritis Res. Ther. 15 (6), R212. 10.1186/ar4407 24325951 PMC3978923

